# Lung Neutrophilic Recruitment and IL-8/IL-17A Tissue Expression in COVID-19

**DOI:** 10.3389/fimmu.2021.656350

**Published:** 2021-03-30

**Authors:** Marina Luise Viola Azevedo, Aline Cristina Zanchettin, Caroline Busatta Vaz de Paula, Jarbas da Silva Motta Júnior, Mineia Alessandra Scaranello Malaquias, Sonia Mara Raboni, Plínio Cezar Neto, Rafaela Chiuco Zeni, Amanda Prokopenko, Nícolas Henrique Borges, Thiago Mateus Godoy, Ana Paula Kubaski Benevides, Daiane Gavlik de Souza, Cristina Pellegrino Baena, Cleber Machado-Souza, Lucia de Noronha

**Affiliations:** ^1^Laboratory of Experimental Pathology, Postgraduate Program of Health Sciences, School of Medicine, Pontifícia Universidade Católica do Paraná, Curitiba, Brazil; ^2^Postgraduate Program in Biotechnology Applied to Child and Adolescent Health, Faculdades Pequeno Príncipe, Instituto de Pesquisa Pelé Pequeno Príncipe, Curitiba, Brazil; ^3^Hospital Marcelino Champagnat, Postgraduate Program of Health Sciences, School of Medicine, Pontifícia Universidade Católica do Paraná, Curitiba, Brazil; ^4^Virology Laboratory, Universidade Federal do Paraná, Hospital de Clínicas, Curitiba, Brazil

**Keywords:** SARS-CoV-2, influenza A virus, H1N1 subtype, interleukin-8, interleukin-17A, neutrophil, polymorphism, immunohistochemistry

## Abstract

The new SARS-CoV-2 virus differs from the pandemic Influenza A virus H1N1 subtype (H1N1pmd09) how it induces a pro-inflammatory response in infected patients. This study aims to evaluate the involvement of SNPs and tissue expression of IL-17A and the neutrophils recruitment in *post-mortem* lung samples from patients who died of severe forms of COVID-19 comparing to those who died by H1N1pdm09. Twenty lung samples from patients SARS-CoV-2 infected (COVID-19 group) and 10 lung samples from adults who died from a severe respiratory H1N1pdm09 infection (H1N1 group) were tested. The tissue expression of IL-8/IL-17A was identified by immunohistochemistry, and hematoxylin and eosin (H&E) stain slides were used for neutrophil scoring. DNA was extracted from paraffin blocks, and genotyping was done in real time-PCR for two *IL17A* target polymorphisms. Tissue expression increasing of IL-8/IL-17A and a higher number of neutrophils were identified in samples from the H1N1 group compared to the COVID-19 group. The distribution of genotype frequencies in the *IL17A* gene was not statistically significant between groups. However, the G allele (GG and GA) of rs3819025 was correlated with higher tissue expression of IL-17A in the COVID-19 group. SARS-CoV-2 virus evokes an exacerbated response of the host’s immune system but differs from that observed in the H1N1pdm09 infection since the IL-8/IL-17A tissue expression, and lung neutrophilic recruitment may be decreased. In SNP rs3819025 (G/A), the G allele may be considered a risk allele in the patients who died for COVID-19.

## Introduction

At the end of 2019, a new strain of the respiratory coronavirus (SARS-CoV-2) emerged from China and has spread rapidly worldwide, causing a wave of infections and deaths (1).

With the rapid increase in the number and severity of COVID-19 cases, ICU professionals have continuously been challenged with the increasing number of patients requiring intensive care, overcrowded hospitals, and limited clinical staff capacity (2–4).

Patients with severe forms of SARS-CoV-2 infection may have acute respiratory distress syndrome (ARDS) with diffuse alveolar damage (DAD). The injury pattern of the infection seems to be a consequence of a complement system activation described as cytokine storm (5). This process is mediated by the angiotensin-converting enzyme 2 (ACE-2) in lung tissue (6, 7), but how this damage starts and compromises the lung tissue needs to be better investigated. Diffuse alveolar damage is associated with a host defense response regulated by a complex interaction of cytokines and inflammatory cells trigger by macrophages activation that can recruit circulating neutrophils, amplifying the response (8–12).

The pulmonary inflammatory reaction to infectious agents, in general, begins with a Th1/Th17 response, producing IL-17 and stimulating pro-inflammatory cytokines, such as IL-8, with consequence increasing vascular permeability allowing the intense neutrophilic infiltrates to give the first combat in viral infection (12).

Genetic variations may be associated with different host responses, and polymorphisms may help understand the host variability response. The single nucleotide polymorphisms (SNPs) can modify protein expression, impacting gene expression. Genotype-phenotype association studies could provide scientific evidence that some SNPs may have a relevant role in common diseases’ pathophysiology (9, 13). Some SNPs for *IL17* are widely studied and associated with infectious pulmonary diseases, determining greater or lesser susceptibility to the disease’s development (14, 15).

This study investigates the involvement of SNPs and tissue expression of IL-17A and the neutrophils recruitment in *post-mortem* lung samples from patients who died of severe forms of COVID-19 comparing to those who died by H1N1pdm09. The results were also compared to *post-mortem* lung samples from control patients.

## Materials and Methods

### Ethical Approvals

The presented study was approved by the National Research Ethics Committee (Conselho Nacional de Ética em Pesquisa—CONEP), protocol number 3.944.734/2020, and 2.550.445/2018.

The authors confirm that all methods were carried out following relevant guidelines and regulations.

Families permitted the *post-mortem* biopsy of the cases of COVID-19, H1N1pdm09, and CONTROL groups; and signed the informed consent forms.

The sample collection followed all relevant ethics and safety protocols.

The data that support the findings of this study are available from the corresponding author upon reasonable request.

### Samples

The COVID-19 group comprises *post-mortem* lung samples of patients whose cause of death was DAD caused by SARS-CoV-2 (n = 20). A minimally invasive lung *post-mortem* biopsy was performed through a left anterior mini-thoracotomy with upper left lobe lingular segment resection. The resected pieces were 3 × 3 cm. Testing for COVID-19 was performed on nasopharyngeal swabs taken during ICU hospitalization, and the performed Real-Time Polymerase Chain Reaction (RT-qPCR). The viral genome’s amplification was performed with the Invitrogen SuperScriptTMIII Platinum^®^ One-Step qRT-PCR Kit (Catalog number: 11732020, Waltham, MA, USA). Clinical data of 20 cases were obtained from medical records during hospitalization in the ICU at Hospital Marcelino Champagnat in Curitiba-Brazil.

The H1N1 group comprises *post-mortem* lung samples (a similar technique to that described for the COVID-19 group) of patients whose cause of death was DAD caused by H1Npdm09 (n = 10) during the 2009 pandemic. The patients were tested through the fresh samples of lung *post-mortem* biopsies, and the performed qRT-PCR (a similar technique to that of the COVID-19 group) was positive for pandemic Influenza A virus H1N1 subtype. Clinical data of 10 cases were obtained from medical records during hospitalization in the ICU at Hospital de Clínicas in Curitiba-Brazil.

The CONTROL group (n = 11) was composed of *post-mortem* lung samples from patients who died of cardiovascular and neoplastic disease, not involving lung lesions.

Survival time was defined as the period between time from hospitalization to death.

### Histological and Immunohistochemistry Analysis

The lung samples provided by *post-mortem* biopsy were formalin-fixed paraffin-embedded (FFPE) and stained with hematoxylin and eosin—H&E (Harris Hematoxylin: NewProv, Cod. PA203, Pinhais, BR; Eosin: BIOTEC Reagentes Analíticos, Cod. 4371, Pinhais, BR).

H&E slides high-resolution images were used to perform the neutrophil scoring in 30 HPF. The scoring was made in hot spot areas, only in the septum or alveolar lumen.

The immunohistochemistry technique was used to analyze IL-8 and IL-17A tissue expression. Anti-IL-17A (rabbit polyclonal antibody, Abcam, Cambridge, UK, Cat# ab91649, RRID: AB_10712684,1:200 dilution) and anti-IL-8 (rabbit polyclonal antibody, Abcam, Cambridge, UK, Cat# ab7747, RRID: AB_306040, 1:200 dilution) were used as the primary antibodies. The validation of the antibodies and the optimal dilution was performed according to the manufacturer’s instructions, using the recommended positive control, in these cases, human samples. The secondary polymer was Reveal Polyvalent HRP-DAB Detection System, Cat# SPD-125, Spring Bioscience, CA, USA. The result was confirmed by positive control’s positivity, a sample known to be positive for a specific antibody allocated together with the patient’s samples.

The immunostained slides were scanned (Axion Scan.Z1 Scanner, Zeiss AG, Oberkochen, Germany), and then ZEN Blue Edition (Zeiss, Germany) was used to randomly generate 10 high-power fields per case (HPF = 40× objective). The analysis was blind once the images were randomly generated by the software, with no investigator’s interference. The image analysis was performed (Image-Pro Plus 4 software). After that, a percentage of IL-8 and IL-17A tissue expression per HPF was obtained in each case.

### Genetic Extraction and Amplification

The DNA was obtained from FFPE cuts of the samples using a commercially available paraffin DNA extraction kit (Qiagen^®^). Once measuring the extracted DNA concentration, these samples were diluted to a final concentration of 20 ng/μl, for working solution, and stored in a freezer at –20°C with restricted access and only allowed to researchers involved in the project or to the technical personnel for them authorized. The *IL17A* gene’s polymorphisms were chosen by criteria of functional relevance in articles published in qualified journals and confirmed in the SNPinfo NIH site (SNPinfo, 2020). Genotype determination was performed for the following *IL17A* polymorphisms: rs2275913 and rs3819025. The purified DNA was amplified by real-time PCR using fluorescent probes (Applied Biosystems 7500 Real-Time PCR System). Genotyping analysis was blind once the samples were anonymized.

### Statistical Analysis

The comparison of two groups concerning quantitative variables was performed using the non-parametric Kruskal-Wallis test. Values of p < 0.05 indicated statistical significance. The nominal variables were expressed in number and frequency and analyzed by Pearson chi-square test and by Fisher exact test. The data were analyzed using the IBM SPSS Statistics v.20.0 software. Armonk, NY, USA: IBM Corp.

## Results

### Patients’ Baseline and Pathological Features

Clinical characteristics of the COVID-19 (n = 20), H1N1 (n = 10), and CONTROL groups are listed in **Table 1**.

**Table 1:** Sociodemographic and clinicopathological characteristics in the study population.

Variables		COVID-19 (n = 20)	H1N1 (n = 10)	CONTROL (n = 11)	*p-*value	
					COVID-19 *vs* H1N1	COVID-19 *vs* CONTROL
Age^*^		70.7 ± 13.0	43.5 ± 13.9	42.3 ± 14.3	<0.001^c^	<0.001^a^
**Gender^†^**	Male	13 (65.0)	8 (80.0)	8 (72.7)	0.398^b^	0.660^b^
	Female	7 (35.0)	2 (20.0)	3 (27.3)		
**Expression of IL-8^§^**		0.89 (0.11–3.81)	4.13 (0.72–9.13)	0.00 (0.00–0.00)	<0.001^a^	<0.001^a^
**Expression of IL-17A^§^**		2.65 (0.00–9.91)	9.73 (4.34–15.44)	0.00 (0.00–0.00)	<0.001^c^	<0.001^a^
**Neutrophil score^§^**		2.5 (1.3–26.9)	16.7 (11.4–25.8)	3.2 (1.8–10.5)	<0.001^a^	0.231
**Mechanical ventilation (days)^§^**		9 (0–36)	1.5 (1–19)	—	0.020^a^	—
**Time from hospitalization to death or survival time (days)^§^**		11.5 (1–39)	1.5 (1–19)	4.0 (1–46)	0.002^a^	0.004^a^

^*^Mean ± Standard Deviation; **^†^**Absolute number (percentage); **^§^**Median (min-max); ^a^Mann-Whitney U; ^b^Pearson’s chi-square; ^c^Shapiro Wilk.

The main histopathological features of COVID-19 and H1N1 groups are shown in **Supplementary Figure 1**.

The COVID-19 group presented proliferative DAD, with type 2 pneumocyte hyperplasia, numerous hyaline membranes, and poor neutrophil recruitment. We observed a variable number of small fibrinous thrombi in small and medium pulmonary arteries following by neutrophilic endotheliitis. Signs of secondary bacterial pneumonia were not observed (9)

The histopathological features of DAD caused by H1N1pdm09 are different than observed in COVID-19 cases. There are fewer hyaline membranes but higher septal and intra-alveolar neutrophils recruitment. There are no significant fibrinous thrombi and neutrophilic endotheliitis. Signs of bacterial coinfections were found in 80% of cases (blood and sputum culture), with the presence of *Haemophilus influezae* (60%), *Streptococcus pneumoniae* (10%), and *Mycoplasma pneumoniae* (10%) being reported in the samples (9).

Regarding the neutrophil score (**Table 1**), the H1N1 group presented statistically higher percentages than the COVID-19 group (p = 0.0011).

### Immunohistochemical Results

The IL-8 and IL-17A tissue expression of the COVID-19 compared with the H1N1 group are shown in **Supplementary Figure 1** and **Table 1**. IL-8 and IL-17A had significantly higher tissue expression in the H1N1 group than COVID-19 (p < 0.001, respectively).

### Genotyping Results

Genotyping analyses of three genetic transmission modes (additive, dominant, and recessive) were performed to understand the biological plausibility better, and no statistical differences for these two polymorphisms were observed.

**Table 2** shows the analysis between IL-17A tissue expression (immunohistochemistry results) with the genetic distribution in three models. The additive model shows the distribution of homozygous and heterozygous genotypes. Dominant shows the grouping homozygous plus heterozygous genotype for wild allele compared to the homozygous genotypes for the polymorphic allele. The recessive model is the reverse (see **Supplementary Tables 1** and **2**).

**Table 2:** IL-17A tissue expression and *IL17A* polymorphisms in all three groups.

Reference SNP^*^ and variation	*IL17A* Genotypes/IL-17A Tissue expression		COVID-19 (n = 20)^†; §^		H1N1 (n = 10)^†; §^		*p*-value^1^	CONTROL (n = 11)^†; §^		*p*-value^2^
			Genotype	Tissue expression	Genotype	Tissue expression		Genotype	Tissue expression	
**rs3819025 G/A**	**Additive model**	**GG**	3	2.9 (2.8–5.4)	0	N/A	0.186^a^	0	N/A	0.170^a^
		**GA**	7	3.2 (1.0–9.9)	2	0.1 (0.0–1.2)		7	0.0 (0.0–1.2)	
		**AA**	9	0.8 (0.0–8.2)	8	0.1 (0.0–1.2)		3	0.0 (0.0–0.0)	
	**Dominant model**	**GG+GAAA**	10	3.2 (1.0–9.9)	2	0.1 (0.0–1.2)	0.090^c^	7	0.0 (0.0–1.2)	0.449^c^
			9	0.8 (0.0–8.2)	8	0.1 (0.0–1.2)		3	0.0 (0.0–1.2)	
	**Recessive model**	**AA+GA**	16	3.3 (0.0–9.9)	10	0.0 (0.0–0.2)	0.532^c^	10	0.0 (0.0–0.2)	0.532^c^
		**GG**	3	2.9 (2.8–5.4)	0	N/A		0	N/A	
									
**rs2275913 G/A**	**Additive model**	**GG**	11	2.9 (0.0–8.2)	5	8.9 (4.3–11.0)	0.355^a^	4	0.0 (0.0–0.0)	0.599^a^
		**GA**	9	1.3 (0.0–9.9)	4	13.3 (5.3–15.4)		5	0.0 (0.0–0.0)	
		**AA**	0	N/A	1	0.00 (0.0–0.0)		0	N/A	
	**Dominant model**	**GG+GA**	20	1.3 (0.0–9.9)	9	10.5 (4.3–15.4)	0.333^c^	9	0.0 (0.0–0.0)	–
		**AA**	0	N/A	1	0.0 (0.0–0.0)		0	N/A	
	**Recessive model**	**AA+GA**	9	1.3 (0.0–9.9)	5	11.9 (5.3–15.4)	0.796^b^	5	0.0 (0.0–0.0)	0.700^c^
		**GG**	11	2.9 (0.0–8.2)	5	8.9 (4.3–11.0)		4	0.0 (0.0–0.0)	

^*^SNP identifier based on NCBI dbSNP; **^§^** IL17A Genotypes were expressed by number; **^†^**Median (Minimum-Maximum) of IL-17A tissue expression. N/A, not applicable; Logistic regression^a^, Pearson’s chi-square^b^, or Fisher’s exact^c^ for ^1^COVID-19 vs. H1N1 and ^2^COVID-19 vs. CONTROL.

rs3819025: The G allele (GG and GA) shows a direct association with higher IL-17A tissue expression in the COVID-19 group than the homozygous A allele. Also, in the H1N1 group, the rs3819025, interestingly, presents shallow tissue expressions of IL-17A, regardless of the models studied.

rs2275913: The genotype GG could be associated with the higher IL-17A tissue expression than GA in the COVID-19 group. The G allele (GG and GA) appears to be associated with this higher IL-17A tissue expression in the H1N1 group. Also, the highest tissue expression for IL-17A was observed in this SNP for the H1N1 group.

Another interesting result is that the IL-17A tissue expression was slightly lower in rs2275913 than rs3819025 for the COVID-19 group and significantly higher in rs2275913 than rs3819025 for the H1N1 group. Finally, for the CONTROL group, the tissue expressions of IL-17A were lowest, regardless of the SNP.

## Discussion

IL-8/IL-17A lung tissue expression and neutrophil score were analyzed to evaluate the Th17 response antiviral, neutrophil-mediated injury, and, consequently, DAD. In response to a viral infection, both IL-8 and IL-17 are secreted by macrophages and T lymphocytes to recruit mainly neutrophils (16). Other studies corroborated these data in patients infected with the H1N1pdm09, which showed increased serological levels of IL-17 (17, 18). H1N1 group features corroborate these findings, as an IL-8/IL-17A tissue expression and neutrophil score increasing were observed. Although neutrophils’ presence is vital to the defense process, their excessive activation can generate severe tissue damage. This pattern was not observed in the COVID-19 group, where IL-8/IL-17A tissue expression and neutrophil score were lower than the H1N1 group (p < 0.001 respectively), indicating that there may be a reduction of Th17 response during the evolution of COVID-19.

A recent study described that, although out of the expected viral lung infection patterns, patients with COVID-19 showed an increased Th2 response. The innate and Th1 responses, mediated by macrophages, neutrophils, and T lymphocytes, are more effective in controlling the viral infection, but this process may be inhibited by reasons that are still unclear SARS-CoV-2 infection (19). The innate and adaptive Th1 response modulation occurs due to viral proteins and the consequent activation of TCD8+ lymphocytes, which may be reduced in infection by SARS-CoV-2. Another current work, dedicated to SARS-CoV-2, reported the immune system’s possible evasion by this virus, explained by the increased incubation time, compared to influenza viruses (19–21).

Under normal conditions, after circulation for 6–12 h, neutrophils undergo apoptosis, but apoptosis is inhibited in the inflammatory state, and the presence of IL-8 can be responsible for this process (13). Disturbances in the innate response mechanism observed in recent studies of SARS-CoV-2 infection may explain the lower expression of IL-8, responsible, among others, for the chemoattraction and neutrophils survival decreased (22, 23).

Studies demonstrate that Influenza A variants can trigger a severe neutrophilic response that appears to correlate with the severity of lung injury and tragic outcomes. It has also been shown that IL-17 and neutrophils chemoattraction may be associated with non-infectious respiratory diseases’ pathogenesis by exacerbating the inflammatory response (24). In this study, the COVID-19 group showed IL-17A/IL-8 lower tissue expression and lower neutrophil score. This result may suggest that the pathogenesis of the SARS-CoV-2 inflammatory response differs from that observed in the H1N1pdm09 infection. One study described neutrophils with possible antiviral potential, and neutrophil depletion observed in SARS-CoV-2 may be associated with the virus immune evasion and viral persistence (25, 26).

Besides, we can also see that both IL-8/IL-17A tissue expression and the neutrophil score remain lower in almost all COVID-19 group patients regardless of time from hospitalization to death (11.5; 1–39), which is higher than the H1N1 group (1.5; 1–19); *p* = 0.002. These differences could explain the neutrophils depletion in this group since the longer-lasting diseases could induce virus immune evasion and burn out of immune response (24–27). Nineteen out of the 20 patients in the COVID-19 group have received mechanical ventilation, and all patients in the H1N1 group underwent this same intensive care procedure. We can also observe that the median time of mechanical ventilation in the COVID-19 group (9; 0–36) was higher than the H1N1 group (1.5; 1–19); *p* = 0.020. Furthermore, COVID-19, unlike H1N1pdm09 patients, underwent gentle mechanical ventilation to avoid the aggravation of the alveolar injury by barotrauma with a consequent increase of cytokines release. With these observations, we may assume that, in this study, perhaps the gentle mechanical ventilation may not aggravate the DAD in COVID-19. However, we must consider that data from *post-mortem* samples reflect only the time of death and fail to predict the disease’s evolution (28).

Authors have suggested that polymorphisms in *IL17A* play a critical role in certain types of cancer, and the rs3819025 has also been studied in different types of disease (29–35). This SNP (rs3819025) is a Single Nucleotide Variation (SNV), and the change is G/A in an intronic region located near exon one in the short arm of chromosome 6 (36). The allele G frequency is 94% in the 1,000 genome database for the European population (36). In our results, the GG genotype (for rs3819025) was present only in the COVID group, and this fact could indicate a risk association for this disease (compared to the H1N1 and CONTROL groups). Besides, the GG/GA genotypes of this SNP are associated with a higher tissue expression of IL-17A in the COVID-19 group. This same GG genotype has been associated with higher serum levels of IL-17A for graft *versus* host disease after allogeneic hematopoietic stem cell transplantation (37). Finally, in the H1N1 group, the tissue expression of IL-17A was very low for this same SNP.

The other SNP analyzed was an SNV 2KB Upstream Variant located in a promoter region (rs2275913 G/A). The allele G frequency is 65% in the 1,000 genome database for the European population (38). The allele G was presented with a higher frequency in all groups. For this polymorphism, the G allele could also be observed associated with the higher tissue expression of IL-17A in both COVID and H1N1 groups. A recent study showed that the G allele’s presence increases infection risk with the influenza A (H1N1) virus (39). Although the study’s focus was COVID-19, our results also indicated an association between rs2275913 and higher IL-17A tissue expression in the H1N1 group.

Despite not having statistical significance in all analyses, when analyzing from the point of view of biological plausibility, it would be reasonable to consider the G allele, both in rs3819025 for COVID-19 group, as in rs2275913 for H1N1 group, a risk allele (compared to the CONTROL group) associated with highest tissue expression of IL-17A.

Our study has some limitations, as the small sample and the autopsy data representing static information at the time of death, and we not reconstructing the disease evolution. We also compare the inflammation process caused by two different viruses, such H1N1pdm09 is an Influenza virus, and SARS-CoV-2 is a coronavirus (28). The authors recognize that to obtain a significant result in genetic association studies, large sample size is required, and, for this reason, the genetic implications are limited. However, the authors believe that this type of study, even with a small sample, could be used in the future to feed databases of systematic reviews and meta-analyzes that could have more power of analysis and thus confirm, or not, these findings.

Corroborating with other authors, and based on our results, SARS-CoV-2 appeared to promote injury by different mechanisms, evading an adaptive Th1 response and inducing a Th2 response, ineffective against viral agents (21, 40–44). In contrast, H1N1pmd09 demonstrated an intense innate response Th1/Th17, responsible for severe lung injury mediated by increased neutrophils recruitment. In both cases, the induction of an exacerbated immune response, either *via* Th1 or Th2, can worsen the clinical condition and poor outcome. Besides, *IL17A* genotyping demonstrated that the G allele of rs3819025 (G/A) might be considered a risk allele in COVID-19 patients and associated with the highest IL-17A tissue expression. This main genetic result should be interpreted with caution, and further studies with a larger sample size may, or not, confirm our findings. However, the findings that involve the presence of risk-associated alleles, for example, could in the future identify individuals more susceptible to the worst outcome when infected with SARS-CoV-2. Finally, our findings could help search for new therapies targeted at decreasing viral replication or blocking inflammatory response.

## Data Availability Statement

The raw data supporting the conclusions of this article will be made available by the authors, without undue reservation.

## Ethics Statement

The studies involving human participants were reviewed and approved by National Research Ethics Committee (Conselho Nacional de Ética em Pesquisa—CONEP), protocol numbers 3.944.734/2020 and 2.550.445/2018. The patients/participants provided their written informed consent to participate in this study.

## Author Contributions

MA performed the immunohistochemistry, was responsible for analysis and interpretation of data, and drafted the manuscript. AZ supported the experiments, including the genotyping analysis. CV supported the experiments, including the immunohistochemistry reactions. JM was responsible for the collection of SARS-CoV-2 patients’ samples and medical records. MM analyzed the data and contributed to the writing of the manuscript. SR was responsible for the collection and preparation of H1N1pmd09 patients’ samples. PN, RZ, AP, NB, TG, AB, and DD are graduate students and provided operational support. NB produced the graphical abstract. CB, CM-S, and LD supervised the project and were significant contributors to the manuscript review. All authors contributed to the article and approved the submitted version.

## Funding

This work was supported by productivity research level 2 of the National Council for Scientific and Technological Development (CNPq). Research financed by Pontifícia Universidade Católica do Paranáa with resources from BRDE- Banco Regional de Desenvolvimento do Extremo Sul.

## Conflict of Interest

The authors declare the research was conducted in the absence of any commercial or financial relationships that could be construed as a potential conflict of interest.

## Supplementary Material

Supplementary Figure 1 Lung sample H&E stained (COVID-19 patient) showing alveolar spaces (black circle) with numerous hyaline membranes (black asterisk) and scarce neutrophilic exudation in the alveolar septa (black arrow). The H1N1pmd09 lung sample shows scarce hyaline membranes (black asterisk) in the alveolar spaces (black circle) and numerous neutrophils in the alveolar septa (black arrow). Lung samples of both groups (COVID-19 and H1N1) showing septal lymphocytes expressing IL-8 and IL-17A (black arrow), scarce in the COVID-19 lung sample and numerous in the H1N1pdm09. In H1N1pdm09 samples is observed numerous lymphocytes expressing IL-17A intermixed with numerous neutrophils and macrophages.

Supplementary Figure 2 Graphical abstract showing the neutrophil recruitment triggered by IL-8/IL-17A.
