# Supplementary figures and images for: Lung Neutrophilic Recruitment and IL-8/IL-17A Tissue Expression in COVID-19
Source: Front Immunol. 2021 Mar 30;12:656350. doi: 10.3389/fimmu.2021.656350 (PMC8044579; doi:10.3389/fimmu.2021.656350)

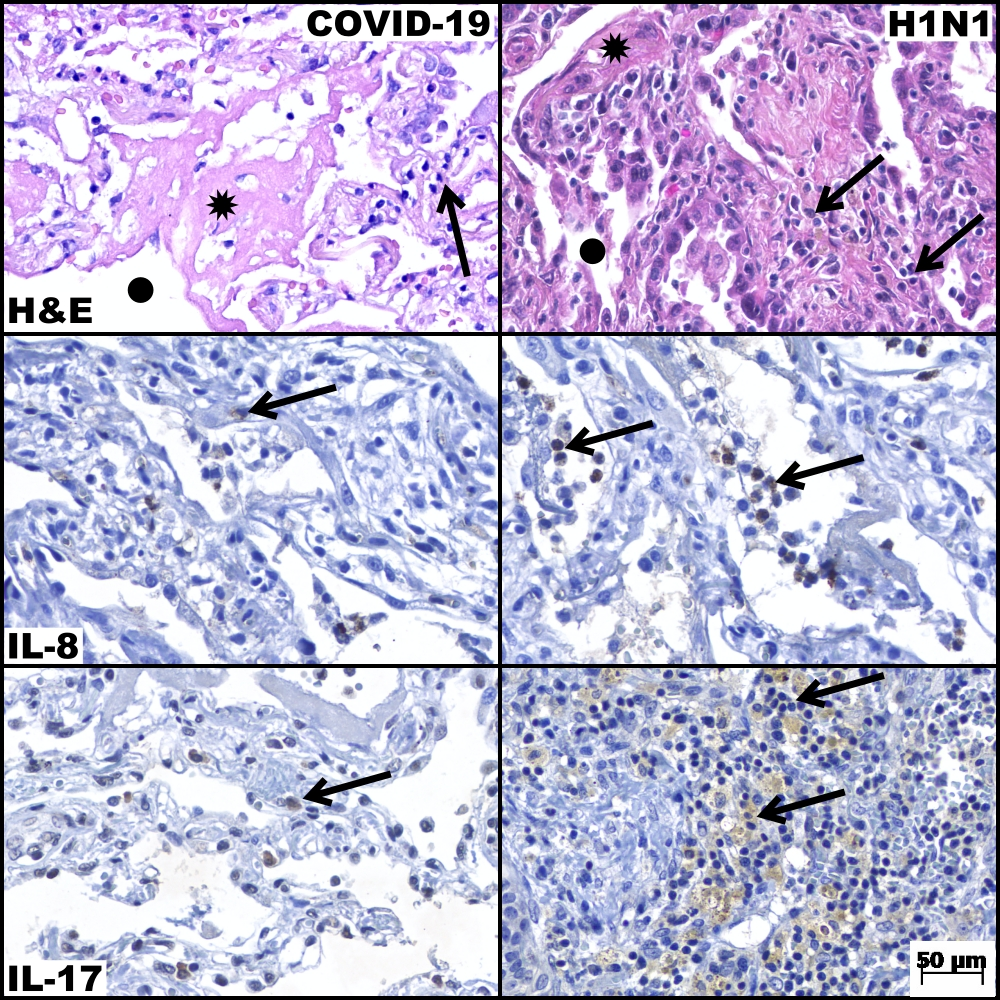

Supplement: Supplementary Figure 1 — Lung sample H&E stained (COVID-19 patient) showing alveolar spaces (black circle) with numerous hyaline membranes (black asterisk) and scarce neutrophilic exudation in the alveolar septa (black arrow). The H1N1pmd09 lung sample shows scarce hyaline membranes (black asterisk) in the alveolar spaces (black circle) and numerous neutrophils in the alveolar septa (black arrow). Lung samples of both groups (COVID-19 and H1N1) showing septal lymphocytes expressing IL-8 and IL-17A (black arrow), scarce in the COVID-19 lung sample and numerous in the H1N1pdm09. In H1N1pdm09 samples is observed numerous lymphocytes expressing IL-17A intermixed with numerous neutrophils and macrophages. [file Image_1.tiff]

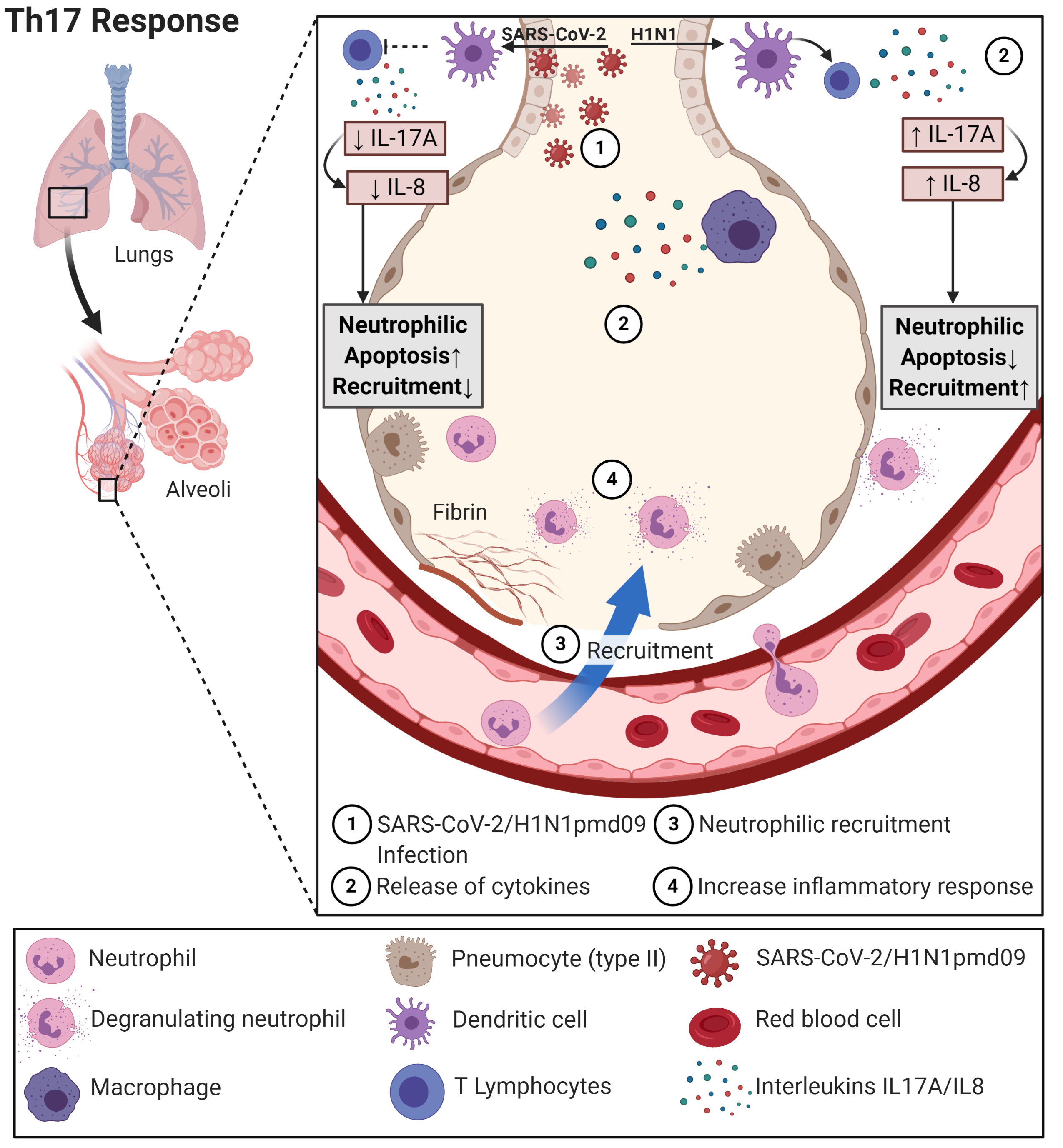

Supplement: Supplementary Figure 2 — Graphical abstract showing the neutrophil recruitment triggered by IL-8/IL-17A. [file Image_2.tiff]
