# Supplementary material for: Lung Neutrophilic Recruitment and IL-8/IL-17A Tissue Expression in COVID-19
Source: Front Immunol. 2021 Mar 30;12:656350. doi: 10.3389/fimmu.2021.656350 (PMC8044579; doi:10.3389/fimmu.2021.656350)
Supplement: Supplementary file 3 [file Table_1.docx]

**Table 1.** Genotypic analysis between 3 groups (COVID, H1N1 and CONTROL) in the *IL17A* gene for addictive model.

| **Gene, reference SNP^*^ and variation** | **Groups** | **Homozygous 1/1** | **Heterozygous 1/2** | **Homozygous 2/2** | ***p*-value^†^** |
| --- | --- | --- | --- | --- | --- |
| ***IL17A***  **rs3819025**  **[G/A]** |  | **GG** | **AG** | **AA** |  |
|  | **COVID-19** | 3 (100) | 7 (43.7) | 9 (45.0) | 0.186^a^ |
|  | **H1N1** | 0 (00.0) | 2 (12.6) | 8 (40.0) |  |
|  | **CONTROL** | 0 (00.0) | 7 (43.7) | 3 (15.0) | 0.170^b^ |
|  |  |  |  |  |  |
| ***IL17A***  **rs2275913**  **[G/A]** |  | **GG** | **AG** | **AA** |  |
|  | **COVID-19** | 11 (55.0) | 9 (50.0) | 0 (00.0) | 0.355^a^ |
|  | **H1N1** | 5 (25.0) | 4 (22.2) | 1 (100) |  |
|  | **CONTROL** | 4 (20.00) | 5 (27.8) | 0 (00.0) | 0.599^b^ |

**Legend:** Genotype was expressed by number and percentage and a total percentage was show in column; **^*^**SNP identifier based on NCBI dbSNP; **^†^** Logistic regression for ^a^COVID-19 *vs* H1N1 and ^b^COVID-19 *vs* CONTROL.

**Table 2.** Genotypic frequency for tag SNPs in the *IL17A* gene in dominant and recessive models.

| **Gene, reference SNP^*^ and variation** | **Models** | **Model** | **COVID-19** | **H1N1** | ***p*-value^a^** | **CONTROL** | ***p*-value^b^** |
| --- | --- | --- | --- | --- | --- | --- | --- |
| ***IL17A***  **rs3819025 [G/A]** | **Dominant G** | **GG+AG** | 10 (52.7) | 2 (10.5) | 0.090**^§^** | 7 (36.8) | 0.449**^§^** |
|  |  | **AA** | 9 (45.0) | 8 (40.0) |  | 3 (15.0) |  |
|  |  |  |  |  |  |  |  |
|  | **Recessive G** | **AA+AG** | 16 (44.4) | 10 (27.8) | 0.532**^§^** | 10 (27.8) | 0.532**^§^** |
|  |  | **GG** | 3 (100.0) | 0 |  | 0 |  |
|  |  |  |  |  |  |  |  |
| ***IL17A***  **rs2275913 [G/A]** | **Dominant G** | **GG+AG** | 20 (52.6) | 9 (23.7) | 0.333**^§^** | 9 (23.7) | 0.719**^§^** |
|  |  | **AA** | 0 | 1 (100.0) |  | 0 |  |
|  |  |  |  |  |  |  |  |
|  | **Recessive G** | **AA+AG** | 9 (47.4) | 5 (26.3) | 0.796**^†^** | 5 (26.3) | 0.700**^§^** |
|  |  | **GG** | 11 (55.0) | 5 (25.0) |  | 4 (20.0) |  |

**Legend:** *IL17A* Genotypes were expressed by number and percentage and a total percentage was show in line; ^*^SNP identifier based on NCBI dbSNP; **^†^** Pearson’s chi-square or **^§^**Fisher’s exact for ^a^COVID-19 *vs* H1N1 and ^b^COVID-19 *vs* CONTROL.
